# Predicting the nature of pleural effusion in patients with lung adenocarcinoma based on ^18^F-FDG PET/CT

**DOI:** 10.1186/s13550-021-00850-2

**Published:** 2021-10-15

**Authors:** Yi Li, Wei Mu, Yuan Li, Xiao Song, Yan Huang, Lei Jiang

**Affiliations:** 1 Department of Nuclear Medicine, Shanghai Pulmonary Hospital, School of Medicine, Tongji University, 507 Zhengmin Road, Shanghai, 200344 China; 2 Beijing Advanced Innovation Center for Big Data-Based Precision Medicine, School of Engineering Medicine, Beihang University, Beijing, 100191 China; 3 Key Laboratory of Molecular Imaging, Chinese Academy of Sciences, Beijing, 100190 China; 4 Department of Thoracic Surgery, Shanghai Pulmonary Hospital, School of Medicine, Tongji University, Shanghai, 200344 China; 5 Department of Pathology, Shanghai Pulmonary Hospital, School of Medicine, Tongji University, Shanghai, 200344 China

**Keywords:** ^18^F-FDG, PET/CT, Pleural effusion, Lung adenocarcinoma, Predictive model

## Abstract

**Background:**

This study aims to establish a predictive model on the basis of ^18^F-FDG PET/CT for diagnosing the nature of pleural effusion (PE) in patients with lung adenocarcinoma.

**Methods:**

Lung adenocarcinoma patients with PE who underwent ^18^F-FDG PET/CT were collected and divided into training and test cohorts. PET/CT parameters and clinical information in the training cohort were collected to estimate the independent predictive factors of malignant pleural effusion (MPE) and to establish a predictive model. This model was then applied to the test cohort to evaluate the diagnostic efficacy.

**Results:**

A total of 413 lung adenocarcinoma patients with PE were enrolled in this study, including 245 patients with MPE and 168 patients with benign PE (BPE). The patients were divided into training (289 patients) and test (124 patients) cohorts. CEA, SUVmax of tumor and attachment to the pleura, obstructive atelectasis or pneumonia, SUVmax of pleura, and SUVmax of PE were identified as independent significant factors of MPE and were used to construct a predictive model, which was graphically represented as a nomogram. This predictive model showed good discrimination with the area under the curve (AUC) of 0.970 (95% CI 0.954–0.986) and good calibration. Application of the nomogram in the test cohort still gave good discrimination with AUC of 0.979 (95% CI 0.961–0.998) and good calibration. Decision curve analysis demonstrated that this nomogram was clinically useful.

**Conclusions:**

Our predictive model based on ^18^F-FDG PET/CT showed good diagnostic performance for PE, which was helpful to differentiate MPE from BPE in patients with lung adenocarcinoma.

**Supplementary Information:**

The online version contains supplementary material available at 10.1186/s13550-021-00850-2.

## Background

Pleural effusion (PE) is a common and challenging clinical problem, which can be caused by numerous malignant and benign diseases. Malignant pleural effusion (MPE) is often observed in several malignant diseases, such as pleural metastasis secondary to lung cancer and breast carcinomas [1–3]. Lung cancer can be classified into non-small cell lung cancer (NSCLC) and small cell lung cancer, and almost a third of the NSCLC patients present PE when they are initially diagnosed [4]. As the most common subtype of NSCLC, lung adenocarcinoma is prone to cause MPE [5–7]. Lung adenocarcinoma cells have a propensity to obstruct pulmonary vessels, particularly the lymphatics, and finally spread to the pleura with fluid accumulation. Moreover, lung adenocarcinoma is usually located in the peripheral pulmonary zone, easily attached to the adjacent visceral pleural surface, or extended to pleural tissues, resulting in malignant pleural infiltration [7].

The diagnosis of MPE affects the tumor stage, therapeutic methods, and prognosis. Once lung adenocarcinoma cells invade the pleura and cause MPE, patients will be staged as IVA and become unsuitable for curative surgical resection. MPE associated with lung adenocarcinoma is usually a cytological or histological definition through discovering the malignant cells in PE or pleural tissue. Thoracentesis is often the first clinical method to diagnose MPE, but the sensitivity of cytological diagnosis based on the pleural fluid is at most 60% [8]. Moreover, 13.2% of the NSCLC patients with pleural fluid less than the 10-mm thickness on chest computed tomography (CT) or lateral decubitus radiography are technically unsuitable for thoracentesis [9, 10]. Percutaneous or thoracoscopic-guided pleural biopsy can improve the positive detection rate of MPE. However, these procedures are invasive and even failed because of the patients’ physical condition or unqualified specimens [11].

At present, the nature of PE can be differentiated on the basis of the morphological change of the pleura through imaging examinations, among which a chest CT scan is the most popular method. Some CT features, including nodular or massive pleural thickening, intrathoracic lymph node enlargement, chest wall or adjacent muscular tissue involvement, and density change with CT attenuation value in effusion, are highly indicative of pleural metastasis and MPE [12]. However, all these imaging features are not specific, which may lead to false positive results due to tuberculosis, inflammation, or other benign diseases [13].

Fluorine-18-fluorodeoxyglucose (^18^F-FDG) positron emission tomography/CT (PET/CT) is an integrated imaging modality combining the metabolic characteristics of PET and morphologic features of CT and has been widely applied to differentiate benign and malignant diseases. Several studies suggested that PET/CT parameters such as maximum standardized uptake value (SUVmax) of pleural tissue or effusion can be used to identify the nature of PE [13–15]. However, these parameters could not achieve high accuracy in diagnostics [11]. Hence, this study aims to establish a predictive model based on ^18^F-FDG PET/CT parameters and to provide effective guidance for clinicians to differentiate MPE from benign PE (BPE).

## Materials and methods

### Patients

From September 2015 to December 2019, 10,976 patients with lung adenocarcinoma who underwent ^18^F-FDG PET/CT examination in our department were retrospectively analyzed. The details of the exclusion criteria were as follows: (1) patients with no PE detected on PET/CT; (2) patients with single or multiple distant metastases; (3) patients with suspected BPE but fewer than two cytological or histological examinations of pleural fluid or biopsy specimen; (4) patients with suspected BPE but follow-up shorter than 12 months; (5) the interval between pleural cytological or histological examination and PET/CT scan was longer than 4 weeks; (6) patients with prior systemic or local therapy before PET/CT scan; (7) patients with other concomitant cancer or history of cancer; (8) patients with renal insufficiency, hepatic cirrhosis, or heart failure; and (9) patients with insufficient clinical data, such as smoking history and serum tumor markers.

### Diagnostic criteria

All patients underwent tumor resection or puncture biopsy, and hematoxylin–eosin and immunohistochemical staining were conducted for pathological-type confirmation of lung adenocarcinoma. The diagnosis of MPE was established on the basis of malignant cells found in the cytological or histological examination of the pleural fluid or pleura, which was obtained within 4 weeks before or after the ^18^F-FDG PET/CT scan. The diagnosis of BPE must meet the following criteria: (1) malignant cells were not found in the surgical pathology of the pleural fluid and pleura; (2) patients had at least two negative results of cytological or histological examination of the pleural fluid or pleura; and (3) patients were followed up for at least 12 months to ensure the absence of malignant pleural processes [16]. The histopathological and cytological results were reviewed by two experienced pathologists. In cases of discrepancy regarding these results, a consensus was reached after a mutual discussion.

### ^18^F-FDG PET/CT scan

PET/CT scans were performed on a Biograph 64 system (Siemens Healthineers, Erlangen, Germany) with a 22.1-cm axial field of view. The patients were required to fast for at least 6 h before imaging, and serum glucose levels were kept lower than 7.4 mmol/l. Images were captured ~ 60 min after intravenous administration of 3.7 MBq of FDG per kilogram of body weight. CT was performed under the following conditions: 120 kV, 100–200 mA (adjusted by auto mA). PET images were acquired for 2.5 min per bed position from the skull base to the midthighs and reconstructed at 200 × 200 pixels using a Gaussian filter of 5.0 mm full width at half maximum value. All image reconstructions were performed with the ordered subset expectation–maximization algorithm, incorporating a CT-based transmission map.

### ^18^F-FDG PET/CT parameters

The PET/CT imaging results were analyzed and interpreted by two experienced nuclear medicine physicians who were unaware of the patients’ clinical information, other imaging and pathology results. In cases of discrepancy regarding PET/CT findings, a consensus was reached after mutual discussion between them. The PET semiquantitative parameter SUVmax was obtained by a circular region of interest (ROI) with proper diameter, placed manually over the corresponding area in the cross-sectional slice of attenuation-corrected emission images using TrueD software (Siemens, Erlangen, Germany). Pleural thickening was characterized as either focal or diffusely thickening, and the contour of diffusely thickened pleura was defined as smooth or irregular [17, 18]. For pleural tissue, the average SUVmax was derived from 3 to 5 ROIs overlaid onto areas with prominent FDG uptake. For PE, the ROI was placed in the deepest part of the pleural fluid to avoid the effect of pleural tissue. For the hilar lymph node, the ROI was chosen from the hilar side with PE.

PET/CT parameters was as follows: (1) SUVmax of tumor; (2) tumor size; (3) tumor attachment to the pleura: tumor attachment to the visceral pleura on both lung window and mediastinal window of CT images, or the inter-lobar pleura to the adjacent lobe on the lung window [19]; (4) tumor with SUVmax ≥ 2.5 and attachment to the pleura; (5) obstructive atelectasis or pneumonia; (6) pleural thickening ≥ 3 mm: either focal or diffuse pleural thickening more than 3 mm; (7) pleural thickening ≥ 10 mm: either focal or diffuse pleural thickening more than 10 mm; (8) focal pleural thickening ≥ 10 mm: focal pleural thickening more than 10 mm and presented as nodular; (9) diffuse smooth pleural thickening: diffuse and regular pleural thickening [18]; (10) diffuse irregular pleural thickening: diffuse and uneven pleural thickening [18]; (11) SUVmax of pleura; (12) pleural thickening ≥ 3 mm with SUVmax ≥ 2.5; (13) pleural thickening ≥ 10 mm with SUVmax ≥ 2.5; (14) focal pleural thickening ≥ 10 mm with SUVmax ≥ 2.5; (15) diffuse smooth pleural thickening with SUVmax ≥ 2.5; (16) diffuse irregular pleural thickening with SUVmax ≥ 2.5; (17) pleural calcification; (18) unilateral and or bilateral PE; (19) CT attenuation values of PE: expressed in Hounsfield Units (HU); (20) SUVmax of PE; (21) hilar or mediastinal lymph node enlargement: defined as the short-axis diameter of lymph node larger than 10 mm; (22) SUVmax of hilar or mediastinal lymph node; and (23) hilar or mediastinal lymph node enlargement with SUVmax ≥ 2.5.

### Statistical analysis

Statistical analyses were performed using SPSS 25.0 software (Illinois, USA), R version 3.6.2 software (Vienna, Austria), and Medcalc 19.1 software (Ostend, Belgium). An independent t-test was conducted to compare the difference of continuous variables. The Chi-squared test or Fisher’s exact test was used to evaluate the difference of categorical variables in the training and test cohorts. Univariate analysis was performed to identify the significant variables in discriminating MPE and BPE, and only variables with *P* < 0.05 were used in the multivariate logistic regression analysis. The predictive model of PE with the combination of significant variables was devised by the results of the multivariate logistic regression, which presented as the diagnosis discriminant equation. A nomogram was built as a graphic representation of the predictive model for MPE through c-statistics. The receiver operating curve (ROC) was used to determine the optimal cutoff value, which was defined on the basis of the maximum Youden’s index (sensitivity + specificity − 1). Sensitivity, specificity, positive predictive value (PPV), negative predictive value (NPV), and the area under the curve (AUC) were calculated. To test the predictive value, the predictive model was validated in the test cohort. Calibration curves of the nomogram were subjected to bootstrapping with 1000 resamples for the internal validation of the training cohort and external validation of the testing cohort. A Hosmer–Lemeshow test was performed to compare the predicted and actual probability of MPE. Decision curve analysis was performed to evaluate the clinical utility of this model in differentiating MPE from BPE for patients with lung adenocarcinoma, by quantifying the net benefits for a range of threshold probabilities in the combined training and test cohorts. Two-tailed *P* < 0.05 was considered statistically significant.

## Results

### Patients’ characteristics

**Fig. 1:**
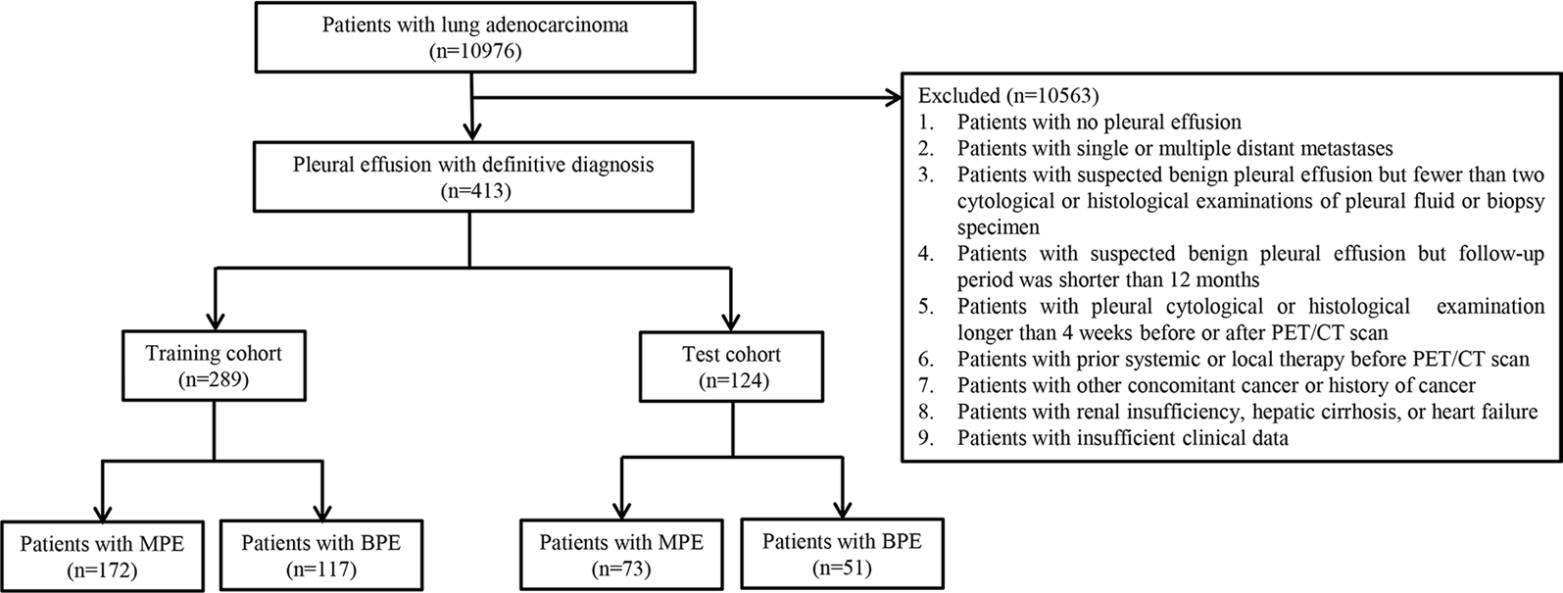
Flowchart of the search strategy and study selection

A total of 413 patients with an average age of 59 ± 12 years (range 20–89 years) were enrolled in this study, comprising 219 men and 194 women. Among the 413 patients, there were 245 patients with MPE and 168 patients with BPE. The causes of BPE included parapneumonic effusion, tuberculosis, pneumosilicosis, and nonspecific pleurisy. All patients were randomly divided into training (289 patients) and test (124 patients) cohorts with a ratio of 7: 3 (Fig. 1). The training cohort was composed of 164 men and 125 women, with an average age of 59 ± 12 years (range 20–89 years), and there were 172 patients with MPE and 117 patients with BPE, respectively. The test cohort consisted of 55 men and 69 women, with an average age of 57 ± 12 years (range 27–81 years), and there were 73 patients with MPE and 51 patients with BPE, respectively.

### ^18^F-FDG PET/CT

For ^18^F-FDG PET/CT results of 413 patients, there were 405 agreements and 8 discrepancies. The overall discrepancy rate was 1.9%. The most frequently contested discrepancy was to distinguish between lung cancer and obstructive atelectasis or pneumonia (*n* = 5), and the other discrepancy was to distinguish between pleural thickening and PE (*n* = 3). For these discrepancies, we discussed and analyzed their ^18^F-FDG PET/CT results, and finally reached agreements.

### Comparison of clinical and ^18^F-FDG PET/CT characteristics in MPE and BPE

Clinical and ^18^F-FDG PET/CT characteristics of the patients included in the training cohort are summarized in Table 1. A total of 16 parameters were found to be significantly different between patients with MPE and those with BPE (*P* < 0.05). Next, ROC analysis was performed to evaluate the diagnostic efficacy of each parameter (Additional file 3: Table 1). For any single parameter, its sensitivity, specificity, PPV, and NPV were not satisfactory.

**Table 1:** Clinical and ^18^F-FDG PET/CT characteristics in the training cohort

Characteristics	MPE (*n* = 172)	BPE (*n* = 117)	*P* Value
Age (mean ± SD, range, years)	60 ± 12 (24–89)	59 ± 13 (20–89)	0.648
*Sex*, *n*			
Male	97	67	0.904
Female	75	50	
*Smoking history*, *n*			
Yes	74	48	0.808
No	98	69	
*CEA *(*ng/mL*), *n*			
> 6.0	91	13	< 0.001*
≤ 6.0	81	104	
*CYFRA211 *(*ng/mL*), *n*			
> 4.0	31	14	0.188
≤ 4.0	141	103	
*NSE *(*ng/mL*), *n*			
> 20	27	11	0.156
≤ 20	145	106	
*PET/CT parameters*			
SUVmax of prime tumor (mean ± SD, range)	8.0 ± 4.8 (2.1–24.9)	6.4 ± 5.7 (0.6–27.7)	0.011*
Tumor size (mean ± SD, range, mm)	36 ± 15 (7–84)	32 ± 15 (6–76)	0.067
Tumor attachment to the pleura	142	42	< 0.001*
Tumor with SUVmax ≥ 2.5 and attachment to the pleura	138	15	< 0.001*
Obstructive atelectasis or pneumonia	78	15	< 0.001*
Pleural thickening ≥ 3 mm	146	84	0.008*
Pleural thickening ≥ 10 mm	122	69	0.043*
Focal pleural thickening ≥ 10 mm	69	42	0.538
Diffuse smooth pleural thickening	17	15	0.450
Diffuse irregular pleural thickening	54	19	0.004*
SUVmax of pleura (mean ± SD, range)	4.2 ± 2.2 (0.9–12.5)	2.5 ± 1.3 (1.0–8.5)	< 0.001*
Pleural thickening ≥ 3 mm with SUVmax ≥ 2.5	130	51	< 0.001*
Pleural thickening ≥ 10 mm with SUVmax ≥ 2.5	113	43	< 0.001*
Focal pleural thickening ≥ 10 mm with SUVmax ≥ 2.5	61	20	0.001*
Diffuse smooth pleural thickening with SUVmax ≥ 2.5	17	13	0.845
Diffuse irregular pleural thickening with SUVmax ≥ 2.5	52	16	0.001*
Pleural calcification	2	4	0.226
Unilateral/bilateral pleural effusion	158 / 14	100 / 17	0.120
CT attenuation value of pleural effusion (mean ± SD, range, HU)	10 ± 5 (0–23)	8 ± 5 (0–23)	0.004*
SUVmax of pleural effusion (mean ± SD, range)	1.7 ± 0.5 (0.6–3.0)	1.2 ± 0.4 (0.1–3.0)	< 0.001*
Hilar or mediastinal lymph node enlargement	127	95	0.158
SUVmax of hilar or mediastinal lymph node (mean ± SD, range)	6.0 ± 4.2 (0.6–20.3)	4.9 ± 4.6 (0.4–24.5)	0.032*
Hilar or mediastinal lymph node enlargement with SUVmax ≥ 2.5	114	75	0.707

*Statistically significant data

### Univariate and multivariate analyses with MPE

Univariate logistic regression analysis showed that the above 16 parameters were also significantly associated with MPE (*P* < 0.05, Table 2). Multivariate logistic regression analysis revealed that abnormal serum CEA levels (OR 11.315, 95% CI 3.456–37.042, *P* < 0.001), tumor with SUVmax ≥ 2.5 and attachment to the pleura (OR 19.729, 95% CI 2.226–174.862, *P* = 0.007), obstructive atelectasis or pneumonia (OR 12.185, 95% CI 3.253–45.648, *P* < 0.001), SUVmax of pleura (OR 2.510, 95% CI 1.547–4.073, *P* < 0.001), and SUVmax of PE (OR 35.305, 95% CI 8.345–149.374, *P* < 0.001) were independent significant factors for predicting MPE in the training cohort (Table 2).

**Table 2:** Univariate and multivariate logistic regression analyses for the diagnosis of MPE in the training cohort

Variables	Univariate analysis			Multivariate analysis		
	*P* value	OR	95% CI	*P* value	OR	95% CI
Abnormal serum CEA levels	< 0.001*	8.988	4.694–17.211	< 0.001*	11.315	3.456–37.042
SUVmax of prime tumor	0.013*	1.064	1.013–1.117	0.082	0.892	0.785–1.014
Tumor attachment to the pleura	< 0.001*	8.452	4.898–14.587	0.491	2.184	0.236–20.219
Tumor with SUVmax ≥ 2.5 and attachment to the pleura	< 0.001*	27.600	14.276–53.359	0.007*	19.729	2.226–174.862
Obstructive atelectasis or pneumonia	< 0.001*	5.643	3.037–10.485	< 0.001*	12.185	3.253–45.648
Pleural thickening ≥ 3 mm	0.007*	2.206	1.235–3.939	0.901	1.159	0.114–11.790
Pleural thickening ≥ 10 mm	0.036*	1.697	1.036–2.782	0.214	0.176	0.011–2.720
Diffuse irregular pleural thickening	0.004*	2.360	1.312–4.247	0.600	3.270	0.039–275.721
SUVmax of pleura	< 0.001*	1.929	1.571–2.370	< 0.001*	2.510	1.547–4.073
Pleural thickening ≥ 3 mm with SUVmax ≥ 2.5	< 0.001*	4.006	2.419–6.634	0.851	1.309	0.078–21.930
Pleural thickening ≥ 10 mm with SUVmax ≥ 2.5	< 0.001*	3.296	2.019–5.382	0.581	2.648	0.083–84.110
Focal pleural thickening ≥ 10 mm with SUVmax ≥ 2.5	0.001*	2.665	1.502–4.731	0.909	1.142	0.119–10.953
Diffuse irregular pleural thickening with SUVmax ≥ 2.5	0.001*	2.735	1.472–5.083	0.633	0.297	0.002–43.162
CT attenuation value of pleural effusion	0.004*	1.073	1.022–1.127	0.171	1.077	0.968–1.198
SUVmax of pleural effusion	< 0.001*	15.488	7.143–33.583	< 0.001*	35.305	8.345–149.374
SUVmax of hilar or mediastinal lymph node	0.034*	1.064	1.005–1.127	0.972	0.998	0.883–1.128

*Statistically significant data

### Prediction model development

The five independent predictive factors identified were entered into multivariate logistic regression analysis with the Enter method, and the results are shown in Table 3. On the basis of the aforementioned logistics analysis, we established the following predictive model to differentiate MPE from BPE: (1) Login (*P*) = *e*^*Y*^/(1 + *e*^*Y*^), and (2) *Y* =  − 9.846 + 2.341 × (abnormal serum CEA levels) + 3.418 × (tumor with SUVmax ≥ 2.5 and attachment to the pleura) + 2.146 × (obstructive atelectasis or pneumonia) + 0.834 × (SUVmax of pleura) + 3.272 × (SUVmax of PE), where “*e*” is the base of the natural logarithm; “abnormal serum CEA levels,” “parenchymal lung lesions with SUVmax ≥ 2.5 and attachment to the pleura,” and “obstructive atelectasis or pneumonia” are 1 if present; otherwise, 0.

**Table 3:** Multivariate logistic regression analysis for independent predictive factors of MPE in the training cohort

Variables	Regression coefficient	*P* value	OR	95% CI
Abnormal serum CEA levels	2.341	< 0.001*	10.394	3.554–30.398
Tumor with SUVmax ≥ 2.5 and attachment to the pleura	3.418	< 0.001*	30.519	10.970–84.905
Obstructive atelectasis or pneumonia	2.146	< 0.001*	8.551	2.891–25.290
SUVmax of pleura	0.834	< 0.001*	2.302	1.654–3.204
SUVmax of pleural effusion	3.272	< 0.001*	26.364	7.737–89.838
Constant	− 9.846	< 0.001*	0.000	

*Statistically significant data

**Fig. 2:**
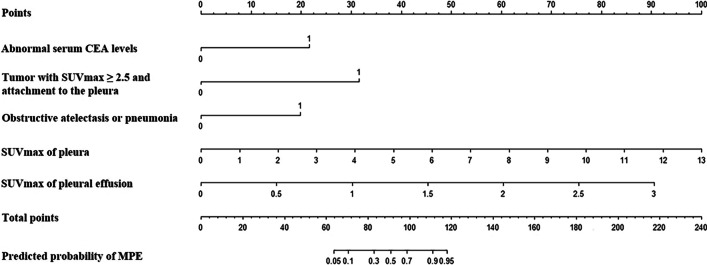
Nomogram predicting the probability of MPE

**Fig. 3:**
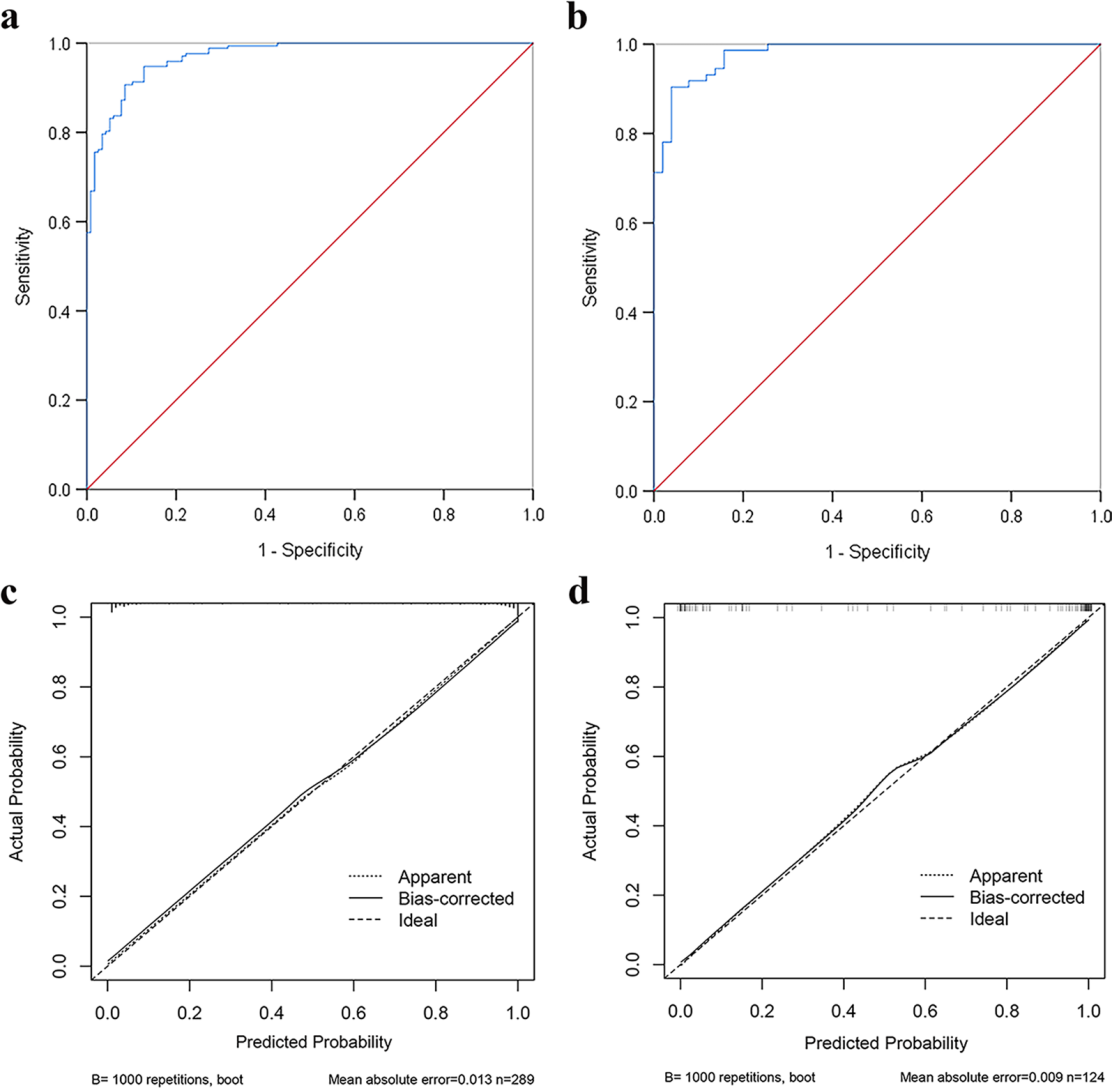
ROC analysis of the predictive model in training (**a**) and test (**b**) cohorts, respectively, and calibration curves of the nomogram in the training (**c**) and test (**d**) cohorts, respectively

This model was further graphically represented as a nomogram shown in Fig. 2. First of all, a response was selected for these independent factors, and a straight line was drawn corresponding to the point. Then, the sum of points from each of these five factors was calculated and labeled as total points. Finally, the individual predictive probability of MPE could be acquired on the basis of the predictive value corresponding to the total points. In the training cohort, this model could achieve an AUC of 0.970 (95% CI 0.954 − 0.986), and the sensitivity, specificity, PPV, and NPV of this predictive model was 90.7%, 91.5%, 94.0%, and 87.0%, respectively, with a cutoff value of 0.641 (Fig. 3a). For example, the cases in Additional files 1 and 2: Figs. 1 and 2 were diagnosed with MPE and BPE based on the predictive model, respectively, which were consistent with cytological or histological examinations. The calibration curves also demonstrated good consistency between predicted and actual probability in the training cohort (Fig. 3c), and the Hosmer–Lemeshow test showed that the chi-square value was 2.733 and *P*-value was 0.950, suggesting no significant departure from the good fit.

### Model test

As shown in Additional files 4 and 5: Tables 2 and 3, 16 parameters were significantly associated with MPE in the test cohort, which was the same as the results in the training cohort. Then, the predictive model was applied to test the diagnostic efficiency in the test cohort. Using the same cutoff as the training cohort, the AUC, sensitivity, specificity, PPV, and NPV in the test cohort could achieve 0.979 (95% CI 0.961–0.998), 91.8%, 90.2%, 93.1%, and 88.5%, respectively (Fig. 3b). Moreover, similar to that in the training cohort, the calibration curve also demonstrated good consistency between predicted and actual probability in the test cohort (Fig. 3d), and the Hosmer–Lemeshow test yielded a chi-square value of 2.729 and a *P-*value of 0.950.

### Clinical use

**Fig. 4:**
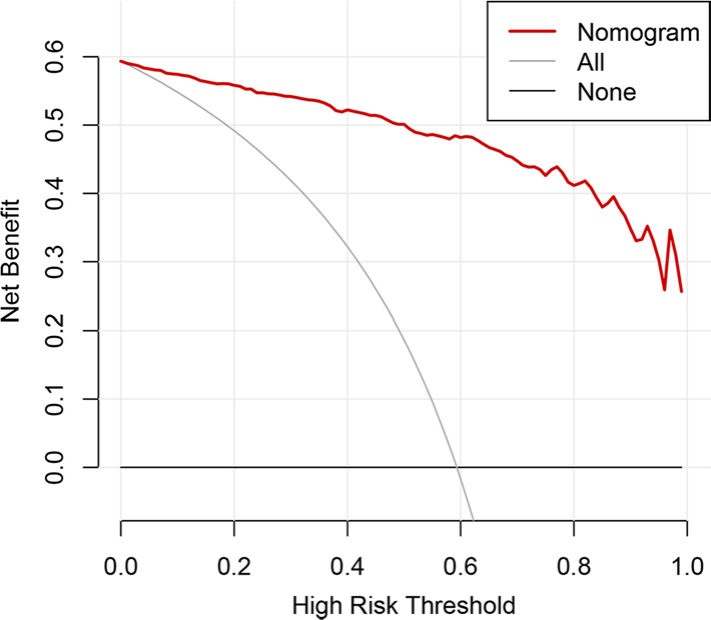
Decision curve analysis for this model in predicting MPE for patients with lung adenocarcinoma. The y-axis measures the net benefit. The grey line represents the assumption that all lesions were malignant (the treat-all scheme). The black line represents the assumption that all lesions were benign (the treat-none scheme). If the threshold probability was more than 5%, using the nomogram to predict malignancy added more benefit than either the treat-all scheme or the treat-none scheme (red line)

Decision curve analysis for predicting MPE based on this nomogram is shown in Fig. 4. If the threshold probability was more than 5%, using the nomogram to predict malignancy added more benefits than either the treat-all scheme (assuming that all PE were malignant) or the treat-none scheme (assuming that all PE were benign). That is, this nomogram achieved the most accurate clinical utility to predict MPE when the threshold probability for a patient is more than 0.05.

## Discussion

Several studies have reported that the parameter series of ^18^F-FDG PET/CT is useful and helpful in distinguishing MPE from BPE, such as glucose uptake of pleura, FDG accumulation of PE, the density of PE, and the morphology of pleura [15, 18, 20]. For example, Kim et al. [20] reported that the increased pleural FDG uptake was the most important parameter for identifying MPE. Nakajima et al. [15] found that the SUVmax of MPE was higher than that of BPE. Sun et al. [18] distinguished MPE from BPE on the basis of pleural glucose metabolism and thickening. However, the performance and change of these parameters are also individually or jointly observed on PET/CT images of patients with BPE, which remains a great clinical challenge.

Based on the previous studies, multiple clinical and ^18^F-FDG PET/CT parameters were investigated and analyzed in this study, revealing that five parameters were significant independent factors for MPE, including abnormal serum CEA levels, tumor with SUVmax ≥ 2.5 and attachment to the pleura, obstructive atelectasis or pneumonia, SUVmax of pleura, and SUVmax of PE. As a common tumor marker, elevated CEA can occur in various malignant tumors, such as colon cancer, gastric cancer, lung cancer, and breast cancer. Lung adenocarcinoma is the most common type of lung cancer with elevated CEA. Usually, elevated CEA is less common in the early stage of lung adenocarcinoma and is more common when the tumor grows to a certain extent, pleural dissemination appears, or distant metastasis occurs [21–23]. As the metabolic parameter of ^18^F-FDG PET/CT, SUVmax is used to diagnose the malignancy and to evaluate the tumor invasion and prognosis. In the present study, although there were significant differences in SUVmax of lung tumor and tumor attachment to the pleura between MPE and BPE, these two parameters were not independent factors for predicting MPE. Only under the conditions of combined lung tumor with the SUVmax ≥ 2.5 and attachment to the pleura, it was the independent predictive factor of MPE. Moreover, when lung adenocarcinoma with obstructive atelectasis or pneumonia occurs, tumor cells may tend to spread along pulmonary vessels to the pleural tissues and cause MPE [24]. Furthermore, similar to the previous studies [15, 18, 20], this study demonstrated that SUVmax of pleura and SUVmax of PE were also important parameters for identifying MPE. It is speculated that FDG may be taken up by tumor cells in the PE, and the degree of FDG accumulation may reflect the number of tumor cells in the PE [15].

Subsequently, a predictive model was established based on these five parameters to distinguish MPE from BPE with good diagnostic performance. The sensitivity and specificity of diagnosing MPE based on this model were 90.7% and 91.5% in the training cohort, respectively. Schaffler et al. [25] defined ^18^F-FDG PET findings as positive if the pleural activity was higher than the mediastinal background activity; then, the sensitivity and specificity of combined FDG PET and CT were 100% and 76%, respectively. Porcel et al. [11] conducted a meta-analysis to judge the accuracy of ^18^F-FDG PET/CT for differentiating MPE from BPE in 14 non-high risks of bias studies with 407 patients with MPE and 232 patients with BPE and found the sensitivity and specificity of integrated PET/CT imaging using semiquantitative parameters for identifying MPE were 81% and 74%, respectively. Yang et al. [16] developed a PET/CT scoring for diagnosing MPE, and the sensitivity and specificity were 83.3% and 92.2%, respectively. Compared with previous studies, this predictive model performed well to distinguish MPE from BPE with high sensitivity and specificity. Additionally, a nomogram was constructed to graphically present the predictive model, thereby providing a convenient and personalized tool to predict the probability of MPE. The calibration curves also demonstrated good consistency between predicted and actual probability in the training cohort. Hence, the model showed good discriminatory ability, calibration, and clinical usefulness.

On the one hand, this predictive model could reduce the number of unnecessary invasive procedures of cytological or histological examinations in patients with possible BPE. On the other hand, the model could recommend thoracentesis, percutaneous, or thoracoscopic biopsy in patients with probable MPE. Additionally, ^18^F-FDG PET/CT imaging can guide the site of puncture or biopsy for these invasive operations and minimize sampling errors. Furthermore, these five parameters were also proven to be independent predictive factors for MPE in the test cohort, which were also used to test the diagnostic efficiency of this predictive model in patients of the test cohort. Similar to the results in the training cohort, the sensitivity and specificity for diagnosing MPE in the test cohort were 91.8% and 90.2%, respectively.

There are also some limitations to this study. First, the stage of lung adenocarcinoma patients with single or multiple distant metastases (IVA or IVB) is the same as, or more advanced than, that of patients with pleural dissemination (IVA) [26]. If a patient with lung adenocarcinoma has distant metastasis, the diagnosis of pleural dissemination and MPE has no effect on the tumor stage and may have little influence on the therapeutic approaches. Thus, lung adenocarcinoma patients with distant metastasis were excluded from this study, and this prediction model may not be suitable for predicting MPE in lung adenocarcinoma patients with distant metastasis. Second, in this study, only SUVmax ≥ 2.5 of ^18^F-FDG PET/CT was selected as the reference of glucose metabolism based on numerous previous studies [27–29]. To obtain a better diagnostic performance, other SUVmax thresholds should be performed and compared in future studies. Third, the tumor markers of PE samples, such as CEA, CYFRA211, and CA199, were reported to play a role in the diagnosis of lung adenocarcinoma associated MPE [5]. Because of the sample size and other factors, the tumor markers of PE were not included in the present study. Fourth, the measurement of the SUVmax of the pleura and PE may be influenced by the respiratory movement and partial volume effect especially in patients with relatively low amounts of pleural fluid. Finally, although this predictive model demonstrated good consistency between predicted and actual probability in both training and test cohorts, a cohort from a different health center should be collected to validate this predictive model.

## Conclusion

We constructed a predictive model based on ^18^F-FDG PET/CT for diagnosing the nature of PE, which showed good diagnostic performance for PE. It was greatly helpful to differentiate MPE from BPE in patients with lung adenocarcinoma.

## Supplementary Information

**Additional file 1**: **Figure 1**. A 61-year-old man with adenocarcinoma in the upper lobe of the left lung was demonstrated on PET/CT imaging (a). This patient presented normal serum CEA levels, tumor with SUVmax of 5.8 (b, thin arrows) and attachment to the pleura, obstructive atelectasis or pneumonia, pleura with SUVmax of 4.7 (d, thick arrows), and pleural effusion with SUVmax of 2.0 (d, triangle arrows). The Login (P) value was calculated as 0.998 and the probability of MPE was more than 90% based on this predictive model. Finally, MPE was confirmed by the thoracentesis.

**Additional file 2**: **Figure 2**. A 44-year-old woman with adenocarcinoma in the lower lobe of the right lung and hilar and mediastinal lymph nodes metastases was shown on PET/CT imaging (a). This patient presented normal serum CEA levels, tumor with SUVmax of 13.3 (b, thin arrows) and attachment to the pleura, no obstructive atelectasis or pneumonia, pleura with SUVmax of 1.7 (c, thick arrows), and pleural effusion with SUVmax of 1.0 (d, triangle arrows). The Login (P) value was calculated as 0.150 and the probability of MPE was 10-20% based on this predictive model. Finally, BPE was confirmed by the surgical pathology.

**Additional file 3**: **Table 1**. ROC analysis of clinical and ^18^F-FDG PET/CT characteristics in the training cohort.

**Additional file 4**: **Table 2**. Clinical and ^18^F-FDG PET/CT characteristics in the test cohort.

**Additional file 5**: **Table 3**. Univariate logistic regression analyses for the diagnosis of MPE in the test cohort.

## Data Availability

The datasets used and/or analyzed during the current study are available from the corresponding author on reasonable request.

## Abbreviations

AUC: Area under the curve
BPE: Benign pleural effusion
CT: Computed tomography
^18^F-FDG: Fluorine-18-fluorodeoxyglucose
MPE: Malignant pleural effusion
NPV: Negative predictive value
NSCLC: Non-small cell lung cancer
PE: Pleural effusion
PET/CT: Positron emission tomography/computed tomography
PPV: Positive predictive value
ROC: Receiver operating curve
ROI: Region of interest
SCLC: Small cell lung cancer
SUVmax: Maximum standardized uptake value
