# Supplementary figures and images for: Predicting the nature of pleural effusion in patients with lung adenocarcinoma based on 18F-FDG PET/CT
Source: EJNMMI Res. 2021 Oct 15;11:108. doi: 10.1186/s13550-021-00850-2 (PMC8519982; doi:10.1186/s13550-021-00850-2)

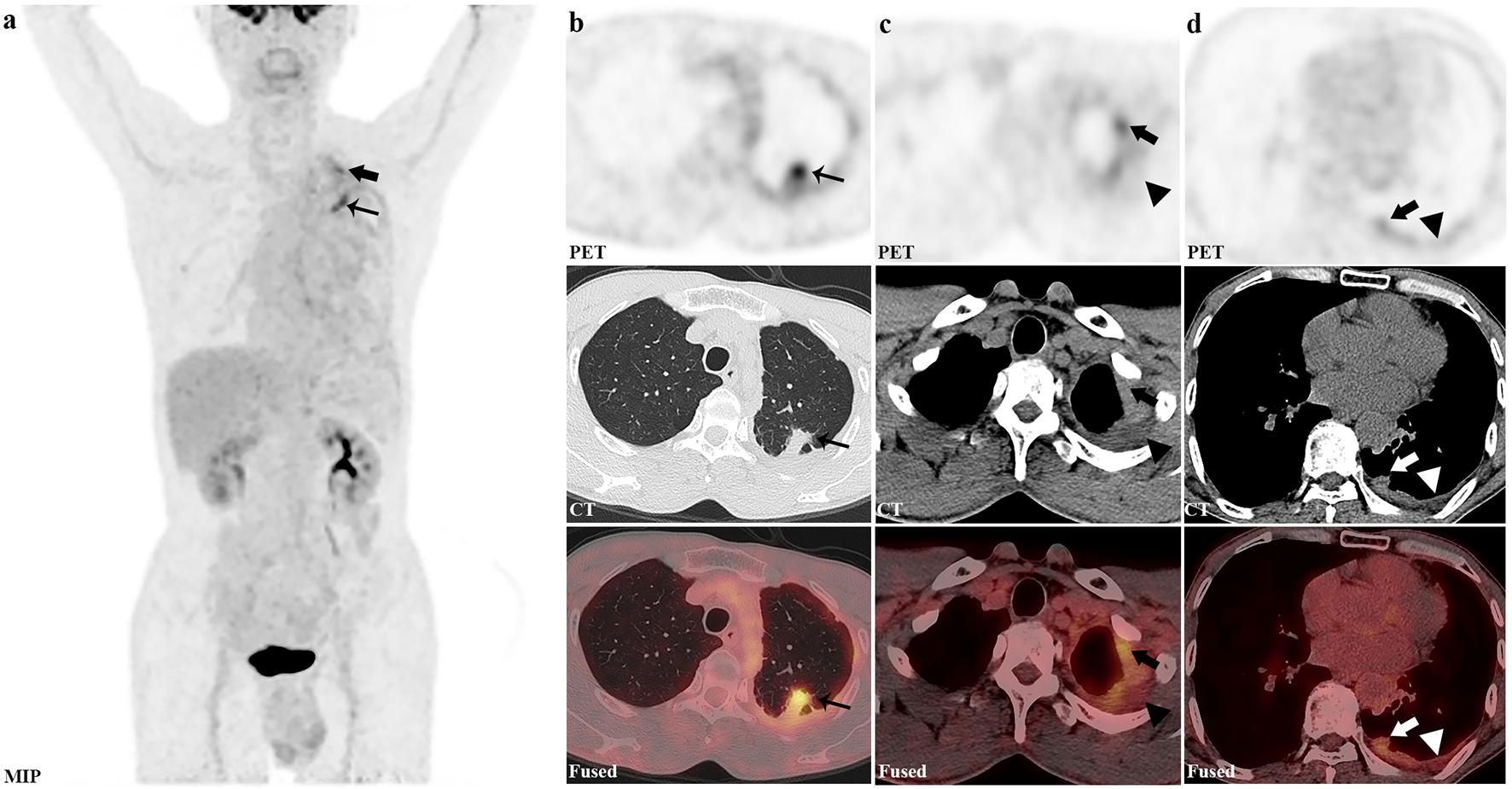

Supplement: Supplementary file 1 — Additional file 1: Figure 1. A 61-year-old man with adenocarcinoma in the upper lobe of the left lung was demonstrated on PET/CT imaging (a). This patient presented normal serum CEA levels, tumor with SUVmax of 5.8 (b, thin arrows) and attachment to the pleura, obstructive atelectasis or pneumonia, pleura with SUVmax of 4.7 (d, thick arrows), and pleural effusion with SUVmax of 2.0 (d, triangle arrows). The Login (P) value was calculated as 0.998 and the probability of MPE was more than 90% based on this predictive model. Finally, MPE was confirmed by the thoracentesis. [file 13550_2021_850_MOESM1_ESM.tif]

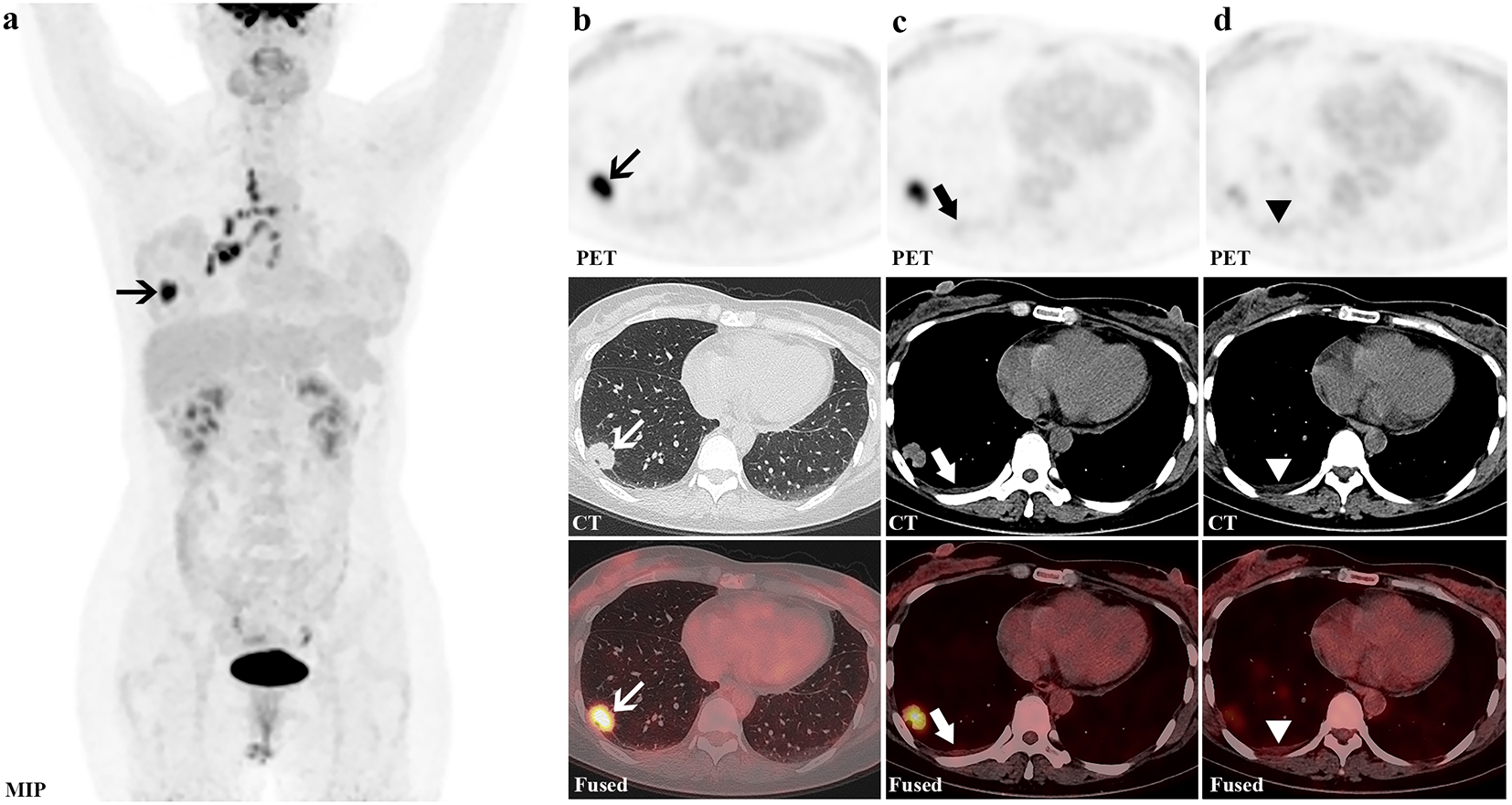

Supplement: Supplementary file 2 — Additional file 2: Figure 2. A 44-year-old woman with adenocarcinoma in the lower lobe of the right lung and hilar and mediastinal lymph nodes metastases was shown on PET/CT imaging (a). This patient presented normal serum CEA levels, tumor with SUVmax of 13.3 (b, thin arrows) and attachment to the pleura, no obstructive atelectasis or pneumonia, pleura with SUVmax of 1.7 (c, thick arrows), and pleural effusion with SUVmax of 1.0 (d, triangle arrows). The Login (P) value was calculated as 0.150 and the probability of MPE was 10-20% based on this predictive model. Finally, BPE was confirmed by the surgical pathology. [file 13550_2021_850_MOESM2_ESM.tif]
