# Supplementary material for: Predicting the nature of pleural effusion in patients with lung adenocarcinoma based on 18F-FDG PET/CT
Source: EJNMMI Res. 2021 Oct 15;11:108. doi: 10.1186/s13550-021-00850-2 (PMC8519982; doi:10.1186/s13550-021-00850-2)
Supplement: Supplementary file 3 — Additional file 3: Table 1. ROC analysis of clinical and 18F-FDG PET/CT characteristics in the training cohort. [file 13550_2021_850_MOESM3_ESM.docx]

**Supplemental Table 1**

| **Characteristics** | **Cutoff Value** | **Sensitivity, %** | **Specificity, %** | **PPV, %** | **NPV, %** |
| --- | --- | --- | --- | --- | --- |
| Abnormal serum CEA levels | - | 52.9 | 88.9 | 87.5 | 56.2 |
| SUVmax of prime tumor | 2.8 | 91.3 | 40.2 | 69.2 | 75.8 |
| Tumor attachment to the pleura | - | 82.6 | 64.1 | 77.2 | 71.4 |
| Tumor with SUVmax ≥ 2.5 and attachment to the pleura | - | 80.2 | 87.2 | 90.2 | 75.0 |
| Obstructive atelectasis or pneumonia | - | 45.3 | 87.2 | 83.9 | 52.0 |
| Pleural thickening ≥ 3 mm | - | 84.9 | 28.2 | 63.5 | 55.9 |
| Pleural thickening ≥ 10 mm | - | 70.9 | 41.0 | 63.9 | 49.0 |
| Diffuse irregular pleural thickening | - | 31.4 | 83.8 | 74.0 | 45.4 |
| SUVmax of pleura | 2.9 | 72.7 | 81.2 | 85.0 | 66.9 |
| Pleural thickening ≥ 3 mm with SUVmax ≥ 2.5 | - | 75.6 | 56.4 | 71.8 | 61.1 |
| Pleural thickening ≥ 10 mm with SUVmax ≥ 2.5 | - | 65.7 | 63.2 | 72.4 | 55.6 |
| Focal pleural thickening ≥ 10 mm with SUVmax ≥ 2.5 | - | 35.5 | 82.9 | 75.3 | 46.6 |
| Diffuse irregular pleural thickening with SUVmax ≥ 2.5 | - | 30.2 | 86.3 | 76.5 | 45.7 |
| CT attenuation value of pleural effusion | 11 | 45.9 | 73.5 | 71.8 | 48.0 |
| SUVmax of pleural effusion | 1.5 | 58.7 | 86.3 | 86.3 | 58.7 |
| SUVmax of hilar or mediastinal lymph node | 4.4 | 63.4 | 69.2 | 75.2 | 56.3 |
