# Supplementary material for: Predicting the nature of pleural effusion in patients with lung adenocarcinoma based on 18F-FDG PET/CT
Source: EJNMMI Res. 2021 Oct 15;11:108. doi: 10.1186/s13550-021-00850-2 (PMC8519982; doi:10.1186/s13550-021-00850-2)
Supplement: Supplementary file 4 — Additional file 4: Table 2. Clinical and 18F-FDG PET/CT characteristics in the test cohort. [file 13550_2021_850_MOESM4_ESM.docx]

**Supplemental Table 2**

| **Characteristics** | **MPE**  **(n=73)** | **BPE**  **(n=51)** | ***P* Value** |
| --- | --- | --- | --- |
| **Age (mean ± SD, range, years)** | 59 ± 11 (27-81) | 56 ± 11 (39-80) | 0.099 |
| **Sex, n** |  |  |  |
| Male | 31 | 24 | 0.714 |
| Female | 42 | 27 |  |
| **Smoking history, n** |  |  | 0.865 |
| Yes | 40 | 29 |  |
| No | 33 | 22 |  |
| **CEA (ng/mL), n** |  |  |  |
| > 6.0 | 40 | 3 | < 0.001* |
| ≤ 6.0 | 33 | 48 |  |
| **CYFRA211 (ng/mL), n** |  |  |  |
| > 4.0 | 14 | 8 | 0.812 |
| ≤ 4.0 | 59 | 43 |  |
| **NSE (ng/mL), n** |  |  |  |
| > 20 | 12 | 6 | 0.606 |
| ≤ 20 | 61 | 45 |  |
| **PET/CT Parameters** |  |  |  |
| SUVmax of prime tumor (mean ± SD, range) | 7.2 ± 5.3 (1.9-22.1) | 5.3 ± 3.2 (1.9-15.4) | 0.019* |
| Tumor size (mean ± SD, range, mm) | 37 ± 17 (13-72) | 35 ± 12 (13-65) | 0.406 |
| Tumor attachment to the pleura | 64 | 16 | < 0.001* |
| Tumor with SUVmax ≥ 2.5 and attachment to the pleura | 54 | 7 | < 0.001* |
| Obstructive atelectasis or pneumonia | 35 | 4 | < 0.001* |
| Pleural thickening ≥ 3 mm | 67 | 34 | 0.001* |
| Pleural thickening ≥ 10 mm | 59 | 29 | 0.005* |
| Focal pleural thickening ≥ 10 mm | 37 | 17 | 0.067 |
| Diffuse smooth pleural thickening | 10 | 10 | 0.459 |
| Diffuse irregular pleural thickening | 19 | 5 | 0.036* |
| SUVmax of pleura (mean ± SD, range) | 5.0 ± 2.8 (0.9-18.0) | 2.3 ± 1.9 (0.9-9.2) | < 0.001* |
| Pleural thickening ≥ 3 mm with SUVmax ≥ 2.5 | 64 | 19 | < 0.001* |
| Pleural thickening ≥ 10 mm with SUVmax ≥ 2.5 | 57 | 16 | < 0.001* |
| Focal pleural thickening ≥ 10 mm with SUVmax ≥ 2.5 | 37 | 10 | 0.001* |
| Diffuse smooth pleural thickening with SUVmax ≥ 2.5 | 9 | 5 | 0.777 |
| Diffuse irregular pleural thickening with SUVmax ≥ 2.5 | 18 | 4 | 0.017* |
| Pleural calcification | 1 | 3 | 0.305 |
| Unilateral / Bilateral pleural effusion | 66 / 7 | 49 / 2 | 0.305 |
| CT attenuation value of pleural effusion (mean ± SD, range, HU) | 10 ± 5 (1-22) | 8 ± 5 (0-21) | 0.008* |
| SUVmax of pleural effusion (mean ± SD, range) | 1.9 ± 0.4 (0.5-3.0) | 1.4 ± 0.5 (0.6-2.9) | < 0.001* |
| Hilar or mediastinal lymph node enlargement | 48 | 38 | 0.328 |
| SUVmax of hilar or mediastinal lymph node (mean ± SD, range) | 5.9 ± 5.5 (0.8-21.7) | 4.1 ± 2.8 (0.9-11.5) | 0.017* |
| Hilar or mediastinal lymph node enlargement with SUVmax ≥ 2.5 | 34 | 27 | 0.584 |

* indicated statistically significant data.
