# Supplementary material for: Predicting the nature of pleural effusion in patients with lung adenocarcinoma based on 18F-FDG PET/CT
Source: EJNMMI Res. 2021 Oct 15;11:108. doi: 10.1186/s13550-021-00850-2 (PMC8519982; doi:10.1186/s13550-021-00850-2)
Supplement: Supplementary file 5 — Additional file 5: Table 3. Univariate logistic regression analyses for the diagnosis of MPE in the test cohort. [file 13550_2021_850_MOESM5_ESM.docx]

**Supplementary Table 3**

| **Variables** | **Univariate Analysis** | | |
| --- | --- | --- | --- |
|  | ***P* Value** | **OR** | **95% CI** |
| Abnormal serum CEA levels | < 0.001* | 19.394 | 5.533-67.975 |
| SUVmax of prime tumor | 0.035* | 1.099 | 1.007-1.200 |
| Tumor attachment to the pleura | < 0.001* | 15.556 | 6.232-38.828 |
| Tumor with SUVmax ≥ 2.5 and attachment to the pleura | < 0.001* | 17.865 | 6.884-46.361 |
| Obstructive atelectasis or pneumonia | < 0.001* | 10.822 | 3.533-33.147 |
| Pleural thickening ≥ 3 mm | < 0.001* | 5.583 | 2.017-15.454 |
| Pleural thickening ≥ 10 mm | 0.005* | 3.197 | 1.431-7.144 |
| Diffuse irregular pleural thickening | 0.030* | 3.237 | 1.121-9.350 |
| SUVmax of pleura | < 0.001* | 1.933 | 1.483-2.521 |
| Pleural thickening ≥ 3 mm with SUVmax ≥ 2.5 | < 0.001* | 11.977 | 4.872-29.443 |
| Pleural thickening ≥ 10 mm with SUVmax ≥ 2.5 | < 0.001* | 7.793 | 3.464-17.531 |
| Focal pleural thickening ≥ 10 mm with SUVmax ≥ 2.5 | 0.001* | 4.214 | 1.838-9.661 |
| Diffuse irregular pleural thickening with SUVmax ≥ 2.5 | 0.022* | 3.845 | 1.216-12.160 |
| CT attenuation value of pleural effusion | 0.010* | 1.099 | 1.023-1.181 |
| SUVmax of pleural effusion | < 0.001* | 16.020 | 5.240-48.971 |
| SUVmax of hilar or mediastinal lymph node | 0.035 * | 1.100 | 1.007-1.202 |

* indicated statistically significant data.
